# Redefining Virtual Assistants in Health Care: The Future With Large Language Models

**DOI:** 10.2196/53225

**Published:** 2024-01-19

**Authors:** Emre Sezgin

**Affiliations:** 1 The Abigail Wexner Reseach Institute at Nationwide Children's Hospital Columbus, OH United States; 2 The Ohio State University College of Medicine Columbus, OH United States

**Keywords:** large language models, voice assistants, virtual assistants, chatbots, conversational agents, health care

## Abstract

This editorial explores the evolving and transformative role of large language models (LLMs) in enhancing the capabilities of virtual assistants (VAs) in the health care domain, highlighting recent research on the performance of VAs and LLMs in health care information sharing. Focusing on recent research, this editorial unveils the marked improvement in the accuracy and clinical relevance of responses from LLMs, such as GPT-4, compared to current VAs, especially in addressing complex health care inquiries, like those related to postpartum depression. The improved accuracy and clinical relevance with LLMs mark a paradigm shift in digital health tools and VAs. Furthermore, such LLM applications have the potential to dynamically adapt and be integrated into existing VA platforms, offering cost-effective, scalable, and inclusive solutions. These suggest a significant increase in the applicable range of VA applications, as well as the increased value, risk, and impact in health care, moving toward more personalized digital health ecosystems. However, alongside these advancements, it is necessary to develop and adhere to ethical guidelines, regulatory frameworks, governance principles, and privacy and safety measures. We need a robust interdisciplinary collaboration to navigate the complexities of safely and effectively integrating LLMs into health care applications, ensuring that these emerging technologies align with the diverse needs and ethical considerations of the health care domain.

Virtual assistants (VAs)—mostly voice assistants, chatbots, and dialogue-based interactive applications—have been leading conversational technologies, being used for health care communications and remote monitoring [1-4]. However, their accuracy and reliability in understanding and responding to medical questions have been limitations [5], which have been slowly improving over the years [6]. Large language models (LLMs) offer scalable and customizable solutions for these limitations of VAs. The body of literature demonstrating the capabilities of LLMs in medicine and health care has been growing, and a number of studies benchmarking LLMs’ performance against each other or humans’ medical knowledge, decision-making processes, and empathic responses have been published [7-11]. Furthermore, LLM-based services improve equitable access to information and reduce language barriers via contextual and culturally aware systems [12] and privacy-preserving, local solutions for low-resource settings [13-16]. This research and evidence give a glimpse at the future of personalized VAs.

In the context of mental health and health information–seeking behavior, we investigated the performance of VAs in responding to postpartum depression–related frequently asked questions. The evidence from our two studies (conducted in 2021 with VAs [17] and 2023 with LLMs [18]) provides comparable findings on the 2-year difference in technology; illuminates the evolving roles of artificial intelligence, natural language processing, and LLMs in health care; and shows the promise of a more accurate and reliable digital health landscape.

In our first study in 2021 [17], we investigated the clinical accuracy of Google Assistant, Amazon Alexa, Microsoft Cortana, and Apple Siri voice assistant responses to postpartum depression–related questions. In our second study in 2023 [18], we replicated our research by using LLMs, and the new study showed significant improvements in the accuracy and clinical relevance of responses. Specifically, GPT-4’s responses to all postpartum depression–related questions were more accurate and clinically relevant in contrast to the VAs, of which the proportion of clinically relevant responses did not exceed the 29% reported in our earlier study. In addition, the interrater reliability score for LLMs (GPT-4: κ=1; *P*<.05) was higher than that for VAs (κ=0.87; *P*<.001), underscoring LLMs’ consistency in clinically relevant responses, which is vital to achieve for health care applications. LLMs also recommended consultations with health care providers—a feature that adds an extra layer for safety—which was not observed in our earlier study with VAs. This dramatic improvement suggests a paradigm shift in the capabilities of digital health tools for health information–seeking activities. The high clinical accuracy and reliability of LLMs point toward a promising future for their integration into existing VA platforms. LLMs can offer dynamic adaptability for VAs via custom applications and decentralized LLM architectures [19,20]. Given their capabilities for open-source developments and collaborations, LLMs could serve as cost-effective and inclusive frameworks for collaborative developments (ie, among technology providers, patients, and clinical experts) in fine-tuning and training VAs for specific medical purposes.

The empirical data from our studies, as well as the literature [21], indicate a compelling trajectory toward LLMs being used to potentially improve the clinical and instructional capabilities of conversational technologies. This suggests a shift in our earlier spectrum model for VAs in health care (Figure 1) [22], in which we proposed 4 service levels for a spectrum of VA use that were associated with the risk, value, and impact of VAs. These levels were the “information” (eg, asking Amazon Alexa to start self-care guidance), “assistance” (eg, setting up reminders for medication or self-therapy), “assessment” (eg, identification, detection, prediction with digital biomarkers, and management), and “support” (prescribing, substituting, or supplementing medication and therapy tools) levels. In 2020, the evidence on the utilization of VAs in health care indicated that VAs were at the “information” and “assistance” levels [22]. However, LLMs are opening up opportunities for VAs, potentially toward the “assessment” and “support” levels. As Figure 1 shows, the level of a service and the associated risk, value, and impact of the service can change based on the targeted problems and solutions. A digital health ecosystem represents the ecosystem where we may envision a future of support from VAs enhanced by LLMs with speech interaction and audio-based sensing capabilities [23]. Such enhancements may include quantifying human behavior–related factors beyond VA engagement, such as social engagement, emotions, neurodevelopmental and behavioral health, sleep health (snoring, heart rate, and movements), respiratory symptoms (sneezing and coughing), and motion (gait, exercise, and sedentary behavior).

**Figure 1:**
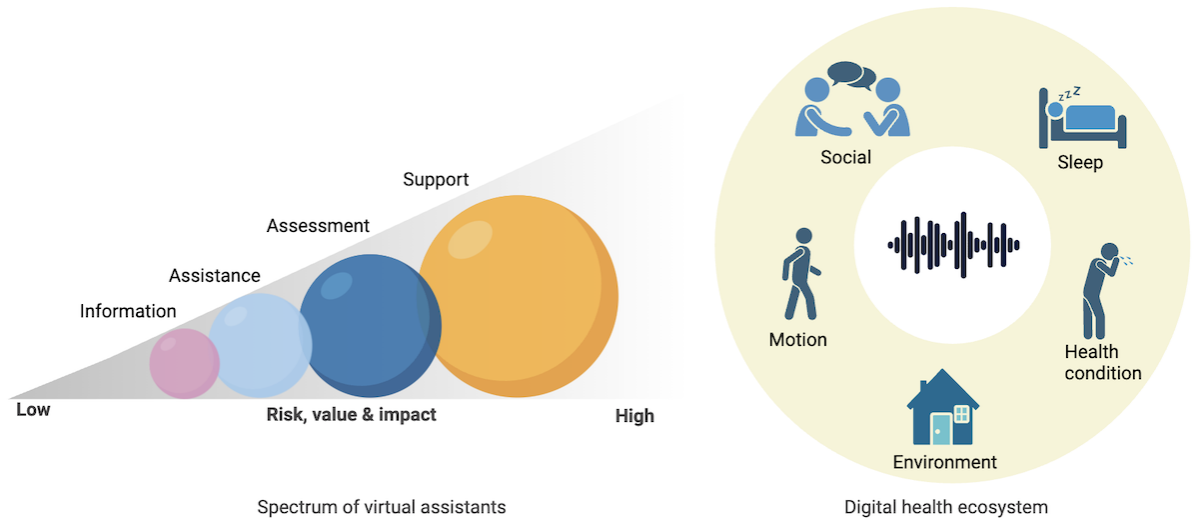
Spectrum of virtual assistants (outlines the risk, value, and impact in health care services) and applications in digital health ecosystems. These can change based on the targeted problems and solutions. This figure was created with BioRender.com (BioRender).

Despite this promising horizon, we need to approach cautiously. As LLMs are becoming highly appealing tools that can be used as VAs in health care, it is imperative to establish a platform that facilitates democratized access and interdisciplinary collaboration during the development of such applications [19,24]. This platform should be designed to bring together a diverse range of stakeholders, including technologists, ethicists, researchers, health care professionals, and patients. This would ensure that the development and integration of VAs are guided by a balanced perspective that considers ethical guidelines and regulatory oversight [25,26], governance principles [27,28], privacy and safety measures [29], feasibility, efficacy, and patient-centric approaches and assessment methods [30,31]. By prioritizing such collaborative and inclusive dialogues, we can better navigate the complex challenges and harness the full potential of these advanced technologies in health care, ensuring that they are developed responsibly, ethically, and in alignment with the diverse needs of all users.

## Abbreviations

LLM: large language model
VA: virtual assistant
